# The experience of pregnant women in contexts of vulnerability of prenatal primary nursing care: a descriptive interpretative qualitative study

**DOI:** 10.1186/s12884-023-05474-z

**Published:** 2023-03-18

**Authors:** Émilie Hudon, Maud-Christine Chouinard, Édith Ellefsen, Jérémie Beaudin, Catherine Hudon

**Affiliations:** 1 Faculté de Médecine et des Sciences de la Santé, Université de Sherbrooke, 3001 12e avenue Nord, Sherbrooke, Québec J1H 5N4 Canada; 2 Département des sciences de la santé, Université du Québec à Chicoutimi, 555 boul. de l’Université Chicoutimi, Chicoutimi, Québec G7H 2B1 Canada; 3 Faculté des sciences infirmières, Université de Montréal, C.P. 6128, succ. Centre-ville, Montréal, Québec H3C 3J7 Canada; 4 Centre de recherche du Centre intégré universitaire de santé et de services sociaux du Nord-de-l’Île-de-Montréal, 5400 boulevard Gouin Ouest, Montréal, Québec H4J 1C4 Canada; 5 École des sciences infirmières, Université de Sherbrooke campus Longueuil, 150 Place Charles-Le Moyne, Longueuil, Québec J4K 0A8 Canada; 6 Département de médecine de famille et de médecine d’urgence, Université de Sherbrooke, 3001 12e avenue Nord, Sherbrooke, Québec J1H 5N4 Canada; 7 Centre de recherche du Centre hospitalier de l’Université de Sherbrooke, 3001 12e Avenue Nord, Sherbrooke, Québec J1H 5N4 Canada

**Keywords:** Pregnant women, Contexts of vulnerability, Prenatal care, Nurse, Experience, Interpretative descriptive approach, Qualitative research, Primary care

## Abstract

**Background:**

Prenatal primary nursing care contributes to improving the health outcomes of mothers and unborn babies. Some pregnant women in contexts of vulnerability experience prenatal nursing care in a positive way, while some do not. A better understanding of factors influencing this experience could help improve prenatal nursing care. The aim of this study was to describe factors influencing the prenatal primary nursing care experience of pregnant women in contexts of vulnerability.

**Methods:**

Thorne’s qualitative interpretative descriptive approach was used. Twenty-four pregnant women in contexts of vulnerability were recruited in local community service centers in Quebec, Canada, using purposive and snowball samplings, to carry out a semi-structured interview. Participants were 16 years old and over, in their second or third trimester, or had given birth in the previous year, and received prenatal nursing care through community health services. Data collection methods included a logbook, sociodemographic questionnaire and semi-structured interview on vulnerable pregnant women’s experience with prenatal primary nursing care. The Qualitative Analysis Guide of Leuven guided the inductive thematic analysis, following a constant comparative iterative process.

**Results:**

The women’s experience was initially influenced by the fulfillment of their needs and expectations. These stem from their previous or current pregnancy experiences, their motivation to receive prenatal care, their family concerns as well as their contexts of vulnerability. From the pregnant women’s perspective, the main factors that influenced their experience were the nurse’s approach, characteristics and interventions that all impact on their relationship with nurses, as well as the prenatal primary care organization, including the modalities of prenatal care (i.e. schedule, setting, duration, number and frequency of meetings), the continuity and the program’s prenatal care services, such as referral to a nutritionist, social worker or other services.

**Conclusions:**

A conceptual framework is proposed to describe relationships among the factors distributed in three dimensions that influence the experience of pregnant women in contexts of vulnerability and to guide nurses in the improvement of prenatal primary care. Considering the complexity of this experience, a person-centered approach is mandatory to promote a positive experience, equity and a better use of services.

**Supplementary Information:**

The online version contains supplementary material available at 10.1186/s12884-023-05474-z.

## Background

A pregnant woman who “is threatened by physical, psychological, cognitive and/or social risk factors in combination with lack of adequate support and/or adequate coping skills” [1, 2], is considered to be in a context of vulnerability [3]. According to the World Health Organization (WHO) [4–6], these contexts contribute to maternal death, stillbirths, prematurity or growth restriction. While, in some countries, midwives may play an important role in prenatal care, in Quebec, Canada, this role is taken by nurses for women in contexts of vulnerability through government programs provided by local community service centers (LCSC). Prenatal nursing care, including, for example, care coordination of care among providers, identification of risks for complications between (e.g. eating disorders or abnormal signs and symptoms during pregnancy), and promotion of healthy lifestyles [7, 8], can improve pregnancy outcomes among women [9] who are likely to underutilize prenatal care, namely by delaying seeking medical attention or having an insufficient number of follow-ups [10, 11]. The primary care nurse can play a key role in prenatal care by promoting maximum utilization of services [9]. Nurse can contribute to the early detection and management of complications [12], in addition to preparing women for childbirth, as well as maintaining and improving health. Specifically, the nurse must support women in contexts of vulnerability by identifying their needs to prepare them for their new role [13].

WHO prenatal care guidelines [6] propose recommendations to improve utilization and quality of prenatal care. While the aim should be the promotion of a positive experience [6], pregnant women’s experience is complex and stems from a subjective interpretation of their prenatal nursing care [14]. A thematic synthesis by our research team [15] aimed to systematically review the literature to describe the prenatal primary nursing care experience of pregnant women in contexts of vulnerability. We observed that women in contexts of vulnerability have needs and expectations throughout their pregnancy. The fulfillment of these needs and expectations shapes their experience and guides their decision to continue, cease or modify their prenatal nursing care. However, their experience may be influenced by some factors not identified in this review. The aim of this study was to describe factors influencing the prenatal primary nursing care experience of pregnant women in contexts of vulnerability. A better understanding of factors influencing this experience could help improve prenatal primary nursing care.

## Methods

### Design

This study used Thorne’s qualitative interpretative descriptive approach (2016) [16]. This approach helps to address a clinical concern in order to improve care experience from the perspective of women in their natural context. Multicenter ethic approval was obtained from the Eastern Townships Integrated University Health and Social Services Center – Sherbrooke University Hospital Ethic Board. The Standards for Reporting Qualitative Research (SRQR) were used to present this study (Additional File 1).

### Sampling methods, setting, and participants

Participants were recruited using a purposive sample method in LCSC (*n* = 21), in Quebec, Canada; a snowball approach (*n* = 1); and through social media (i.e. Facebook) (*n* = 2). These sampling methods allowed us to reach as many pregnant women as possible in contexts of vulnerability. To ensure anonymity and confidentiality, we assigned fictional names to the pregnant women. Generally, prenatal nursing care for women in contexts of vulnerability is provided through the Eggs, milk and orange (Olo [Œufs, lait, orange]), and the Integrated Perinatal and Early Childhood Services (SIPPE [Services intégrés en périnatalité et pour la petite enfance]) programs. While the nurses often meet women at home, they can also organize other appointments at the clinic or by phone. The Olo program provides pregnant women living below the low-income threshold for their region with an equal opportunity to give birth to a healthy child by offering coupons or vouchers for food (i.e. one egg per day, a liter of milk and prenatal multivitamins, plus bag of frozen vegetables per week) [17, 18]. SIPPE is a program provided to inform and support pregnant women who have a low income, who are undereducated or who are socially isolated [19]. This program aims to improve the health status of unborn babies, children and pregnant women.

Prenatal care was provided by a nurse in the women’s home through the Olo and SIPPE programs. Nurses handed out a leaflet explaining the research project to every woman they met. Then, a meeting was planned to conduct an individual interview with the interested women. A consent form was emailed in advance.

### Data collection and trustworthiness

Data were collected by the Principal Investigator between October 2020 and October 2021 through recorded semi-structured interviews lasting approximately 60 min. The women were contacted by telephone or virtually (Zoom) at their convenience. Prior to the interview, the women completed a sociodemographic questionnaire composed of 24 closed-ended questions (Additional File 2). Twenty-four semi-structured interviews were carried out. Data collection ended when redundancy was achieved, that is, when participants did not add new information to the in-depth description of the phenomenon [16, 20].

The Interview Guide (Additional File 3) was developed based on a synthesis of the literature [15] and was adjusted according to the interviews in order to gain a thorough understanding of identified patterns. The Interview Guide helped to collect information on women’s perspective of their prenatal nursing care experience. The interviews were recorded in full.

The Principal Investigator used a logbook to detail each of the project’s steps, including descriptive interview notes, activity notes, notes from the various resource persons, methodological notes, reflexive notes and analytical notes, which provided the investigator with an interpretative view [21]. Reflexive notes helped her take an introspective look at her opinions, beliefs, perceptions and potential biases, including her prenatal nursing care practice, that must be taken into account [22]. The Investigator’s Logbook improves the confirmability and reliability of the findings [23]. The trustworthiness criteria will be discussed from the perspective of Lincoln and Guba (1985) [23] (Table 1).

**Table 1:** Strategies to improve trustworthiness

Trustworthiness criteria	Strategies
*Credibility*	• Recording of interviews with participants • Reflexive journal • Peer debriefing • Integration of verbatim
*Transferability*	• Thick description of the women's context
*Reliability*	• Iterative process of constant benchmarking • Audit Trail • Logbook
*Confirmability*	• Audit Trail • Logbook

### Data analysis

Analysis of the data from the sociodemographic questionnaire and the characteristics of care was carried out with descriptive statistics using NVivo 12 Plus software. Data analysis was conducted in parallel with the interviews to guide subsequent interviews. The transcriptions of interviews were read several times in order to be “immersed in the details” [20]. The Qualitative Analysis Guide of Leuven (QUAGOL) [24, 25] was used to analyze the interviews, a method suited to an interpretative description [26] and to proposing a conceptual framework [24]. The QUAGOL method has two phases, a “paper and pencil” phase and a “qualitative software” phase.

The initial phase helped to create narrative interview report and to represent them in the form of conceptual scheme in order to compare them. During the second phase, meaning units were coded using NVivo 12 Plus software, which helped to identify a list of codes. These codes were grouped together in order to reveal any patterns [16]. The iterative process of constant comparative data analysis through inductive and interpretative reasoning enabled the identification of patterns, thus improving credibility [27]. Investigator triangulation and peer debriefing were used throughout the project in accordance with the collaborative approach favored by the QUAGOL method.

## Results

### Participant characteristics

**Fig. 1:**
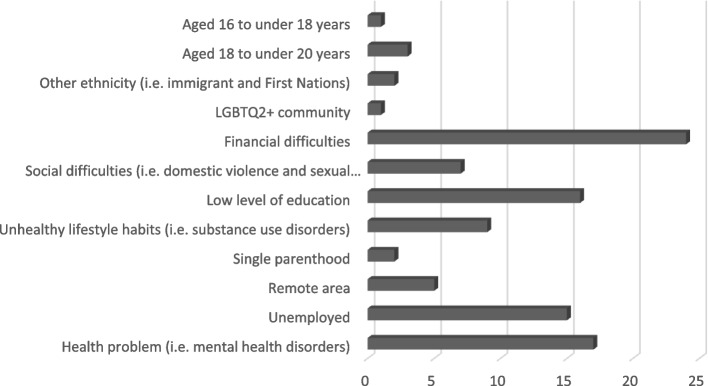
Pregnant women’s contexts of vulnerability Abbreviations. LGBTQ2 + : lesbian, gay, bisexual, transgender, queer (or sometimes questioning), and two-spirited

The average age of participants, 10 pregnant women and 14 women who had given birth in the last 12 months, was 25 years old and all of them were able to communicate in French (Table 2). Half of the participants were primigravida women. All of the women were experiencing different contexts of vulnerability (Fig. 1). Among these, the most common were financial difficulties, lack of employment, the presence of a health problem and a low level of education. Provision of prenatal nursing care began on average at the 12^th^ week of pregnancy. Women had at least 2 to 20 meetings (median [Med] = 9) with one or two nurses. Meetings lasted approximately 60 min, twice a month (Table 3).

**Table 2:** Characteristics of pregnant women in contexts of vulnerability^a^

**Participants (fictitious names)**	***Characteristics of pregnant women in contexts of vulnerability***					
	**Age (in years)**	**Number of weeks**^**days**^** of pregnancy**	**Number of months**^**weeks**^** after delivery**	**Expected fetuses**	**Previous pregnancies**	**Number of follow-ups with Olo/ SIPPE programs including current pregnancy**
*Alya*	22	26 ^0/7^	-	1	0	1
*Aria*	30	24 ^3/7^	-	1	1	1
*Bonita*	37	-	9 ^0^	1	1	1
*Brenda*	16	22 ^4/7^	-	1	0	1
*Camilla*	31	29 ^5/7^	-	1	0	1
*Christina*	23	24 ^0/7^	-	1	1	2
*Clara*	35	-	1 ^3^	1	1	2
*Dalhia*	34	-	9 ^2^	1	2	3
*Ella*	21	30 ^4/7^	-	2	1	2
*Élisa*	20	-	5 ^2^	1	1	1
*Émilia*	21	-	9 ^3^	1	0	1
*Félicia*	30	36 ^5/7^	-	1	1	2
*Fernanda*	18	27 ^4/7^	-	1	0	1
*Flora*	19	21 ^5/7^	-	1	1	2
*Gabriella*	21	-	8 ^2^	1	0	1
*Héléna*	29	-	1	1	1	2
*Isabella*	27	-	1 ^2^	1	0	1
*Jenna*	33	24 ^2/7^	-	1	0	1
*Julia*	20	-	1 ^1^	1	0	1
*Kiara*	24	-	1 ^1^	1	1	2
*Lena*	20	-	8 ^1^	1	0	1
*Lyvia*	26	-	2 ^0^	1	1	2
*Ramona*	38	-	6 ^0^	1	2	3
*Sarah*	19	-	5 ^0^	1	0	1

Abbreviations. *Olo* Eggs, milk and orange corresponding to Œufs-lait-orange, *SIPPE* Integrated Perinatal and Early Childhood Services corresponding to Services intégrés en périnatalité et pour la petite enfance

^a^Dashes (“-”) indicate that the characteristic is not applicable

**Table 3:** Characteristics of prenatal primary nursing care^a^

Participants (fictitious names)	*Characteristics of prenatal primary nursing care*										
	**Total number of meetings**	**Frequency of prenatal follow-up per month**	**Duration of meetings (minutes)**	**Care delivery setting**				**Programs used**		**Number of nurses**	**Number of providers involved in prenatal care**
				***Home***	***LCSC***	***Tel***	***Virtual***	***Olo***	***SIPPE***		
*Alya*	4	1	30–60	x	-	-	-	x	x	1	3
*Aria*	3	1	MD	x	-	-	-	x	x	2	3
*Bonita*	10–15	2	MD	x	x	-	-	x	x	2	4
*Brenda*	5	2	45–60	-	x	-	-	x	x	1	8
*Camilla*	3–4	2	60	-	-	x	-	x	-	1	8
*Christina*	6	2	60–90	x	x	-	-	x	-	1	4
*Clara*	6	1	60	x	-	-	-	x	-	1	4
*Dalhia*	4–5	1	45–90	-	-	x	-	x	x	2	3
*Ella*	10	2	45–60	-	-	x	-	x	x	2	3
*Élisa*	10–15	2	60	-	x	-	-	-	x	1	2
*Émilia*	15	2	15–30	x	-	-	-	-	x	1	2
*Félicia*	10	2	30–45	x	x	-	-	x	x	1	4
*Fernanda*	20	1	MD	x	x	-	-	-	x	1	4
*Flora*	10	2	60	x	-	-	-	x	x	1	4
*Gabriella*	8	2	25–60	x	-	-	-	-	x	1	3
*Héléna*	15	1	30	-	x	-	-	x	x	2	5
*Isabella*	10	2	60	x	-	-	x	x	x	1	4
*Jenna*	2–3	2	30–60	x	-	-	-	x	x	1	4
*Julia*	10–15	2	45–90	x	-	x	-	x	x	1	4
*Kiara*	6–7	2	45–90	x	-	-	-	x	-	2	3
*Lena*	10–15	2	45–60	x	-	x	-	x	x	1	4
*Lyvia*	3	2	30	x	-	-	-	x	x	1	3
*Ramona*	12	2	60	x	-	-	-	x	x	1	3
*Sarah*	10	2	20–40	x	-	-	-	-	x	1	2

Abbreviations. *LCSC* local community service centers, *MD* missing data, *Olo* Eggs, milk and orange corresponding to Œufs-lait-orange, *SIPPE* Integrated Perinatal and Early Childhood Services corresponding to Services intégrés en périnatalité et pour la petite enfance; Tel: telephone

^a^Dashes (“-”) indicate that the characteristic is not applicable

### Factors influencing pregnant women’s experience

From the women’s perspective, fulfillment of their prenatal nursing care expectations and needs influenced their experience. Women’s perception of the nurse and of the prenatal primary care organization also influenced their experience, depending on whether contexts of vulnerability were taken into consideration and whether care was adapted accordingly (Additional File 4).

#### Fulfillment of pregnant women’s prenatal care needs and expectations

Positive past experiences of the services provided by LCSC programs created an expectation of receiving care identical to what the women had experienced in the past, while previous negative experiences raised concerns about future care. As Lyvia expressed, she expected to receive her prenatal nursing care at home because she “was used to it in my first pregnancy”.

Women’s current experience of pregnancy gave rise to expectations and to a need for information related to the pregnancy, breastfeeding, childbirth, the couple and parenthood, prenatal care or continuing with the pregnancy. Family concerns experienced by the fathers as well as behavioral or adaptation problems—prompted by the arrival of a newborn—in the children of women with multiple births could also lead to family-related needs. In Felicia's case, she needed specialized follow-up for her son: “I had concerns with my older child, so [the nurse] referred me to a CLSC educator for his behavioral problems. You know, there are parents who beat their children, but we were the ones who were beaten by our child […]. When Thomas developed anger problems, it was especially with the arrival of COVID, the fact that he was more isolated, with fewer friends, all that, it was a little more complicated, but [the nurse] referred me”.

Some women were motivated by a loved one who recommended prenatal care or by a health care professional who motivated them to use the service: “my mother told me about it because she used it during her first pregnancy and then she liked it” (Sarah); “It was my gynecologist who signed me up” (Bonita). Other pregnant women could be motivated to use prenatal care programs to meet their personal needs and expectations. Among these needs and expectations, the women mentioned needing a resource person with knowledge of prenatal care and to whom they could address their questions, ask for support, and voice their concerns related to the pregnancy or to their life in general.

Needs and expectations stemmed from “problems” and “difficulties”, terms used by women to define their contexts of vulnerability, such as accessing financial hardship vouchers. Women used the following terms to talk about their contexts of vulnerability: “I have a mental health problem”; “I have a low income”; or “I have dysphasia”. Some needs were specific to contexts of vulnerability, such as the need for support for a woman living with cerebral palsy, the need to be reassured during a teen pregnancy (i.e. under 20), the need to talk in a context of isolation or the need for education regarding her partner’s role for a woman from the LGBTQ2 + community.

#### Pregnant women’s perception of nurses

A “caring” approach, where the nurse acted in a frank, open, humorous, reassuring and engaging manner, positively influenced their experience. On the other hand, participants described some nurses as using a colder approach, as mentioned by Helena: “The nurse was not there because it was a passion for her, she was there because she had to be there”. Helena felt like a number. The nurse’s lack of caring or proactivity or the presence of a judgmental attitude negatively affected the women’s experience.

The women mentioned the nurse’s age and experience as having influenced their prenatal care experience. For some women, having a nurse from the same age group helped build their relationship, while other women appreciated having an older nurse. Some women preferred a nurse who had experience, both as a mother and as a professional, to support them. Some women associated the nurse’s young age with inexperience: “She’s not a little 20-year-old who’s never a child, who doesn’t know what she’s doing” (Christina).

The women reported that different nursing interventions were carried out, including maternal and fetal assessment and evaluation, or counselling and education, for example. According to the women, their prenatal nursing care experience was also influenced by the nursing interventions. The same intervention, such as counselling and education, could be perceived as reassuring for some, while it was not for others. Ramona didn’t want to watch a birthing video: “I didn’t feel like being traumatized by watching that”. For her part, Julia was reassured by these videos: “Yes, the delivery stressed me a lot; then, she showed me some videos and it all reassured me”. Therefore, interventions can lead to a positive or a negative experience depending on the woman’s interpretation of it.

These interventions varied from one LCSC to another, from one woman to another, or from one follow-up to another for women with multiple pregnancies. All of the women identified strategies used by the nurse to foster their involvement in prenatal care. Nurses supported the women in managing their health, such as in the management of false labor for an immigrant woman, because, in her culture, she had to go to the birthing center before the onset of contractions. Other nurses explained how to contact other health professionals or access services, often related to their contexts of vulnerability, such as by providing the contact information of a chiropractor who offers services for low-income families. These women felt engaged: “I found it fun […]. I could choose to receive the care or not […]. It wasn’t stressful and it made me feel confident” (Élisa).

#### Pregnant women’s perception of prenatal primary care organization

Each woman had her own preferences in terms of setting, schedule, duration, number of meetings and content. Some women living with anxiety said they were relieved when the meetings took place at home, while others would have preferred meetings at the LCSC as they were not comfortable showing their home. Although none of the women considered the start of the prenatal care to be too early, some felt that it had started too late given their fear of suffering a miscarriage. At the beginning of her pregnancy, Sarah was afraid that she would lose her baby when she had stomach pain or had contractions. Her young age (19 years old) caused her a lot of worry. She would have preferred meetings that were closer together: “At the beginning, it was once a month; I might have enjoyed meeting every two weeks, because I think that during that period, I texted her a lot by email. I had a lot of questions at the beginning”. Sarah did not enjoy her pregnancy because she was always worried: “I didn’t experience this moment as I should have”.

With regard to continuity of care, all of the women preferred to keep the same nurse if they had a good relationship with her. The women were afraid that they would not be comfortable with their new nurse or that continuity of care would be affected. In terms of the available services, the women felt overloaded when they had to meet with several providers. For example, Christina ended her meetings with the nutritionist: “I told them that I was going to skip it because I already had several appointments”. For women who were referred to other services, such as the LCSC nutritionist or community organizations, some were disappointed because they received the service later or not at all. For example, Isabella would have liked the continuity of care “with the social worker to be more regular”. She wanted to meet with the social worker early in her pregnancy, which was not the case. Thus, nurses sometimes offered women the services of other health professionals, but the women did not receive them.

### Conceptual framework

**Fig. 2:**
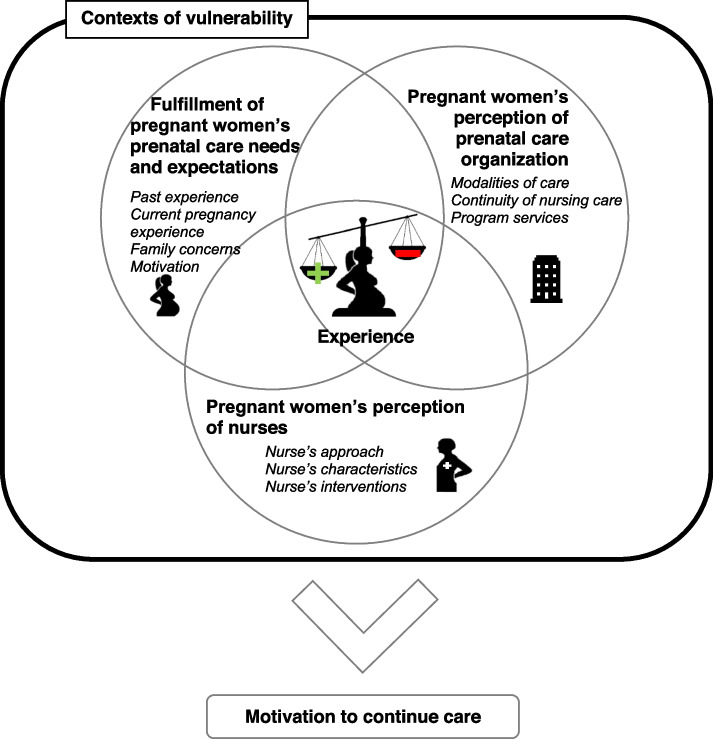
Conceptual framework – Factors influencing the prenatal primary nursing care experience of women in contexts of vulnerability

Figure 2, which is a diagram representing the factors influencing the prenatal nursing care experience of women in vulnerable contexts, illustrates the complexity of the experience. This complexity stems from the cross-influence of the three dimensions, as well as of each factor included in a dimension. The presence of contexts of vulnerability complicates the experience as each woman who is in a particular context can affect each of the factors influencing the experience. In addition, women may be dealing with more than one vulnerability context, thus affecting several factors. For example, Helena had syphilis, a low income, a low level of education (no high school diploma), a daily tobacco habit and follow-ups with youth protection services (YPS), for her other child. She needed reassurance regarding the risks of syphilis affecting the baby, as well as food vouchers and information about smoking cessation. With the prenatal care and the YPS follow-ups, she said she was exhausted by all the meetings with these professionals (i.e. nutritionist, doctor, social worker, high-risk pregnancy clinic, SIPPE nurse).

Therefore, the overall experience of prenatal care translates to a balance between the factors leading to a positive or a negative experience. This experience has an impact on women’s motivation to engage in their care, and thus, to continue using the services. For example, Aria did not enjoy having two nurses intervene in her care, that her family concerns were not taken into account by her first nurse and that having the meetings at her home was imposed on her when she was not comfortable showing her home to others. Nevertheless, she had a “good” experience, as she appreciated the caring attitude of the second nurse, the proactive way in which she met Aria’s needs and expectations, and the fact that they were similar in age, which made the relationship easier according to Aria.

## Discussion

This study documents the factors influencing the prenatal nursing care experience of women in contexts of vulnerability. The results illustrate the complexity of the experience, due to its multidimensionality combined with the cross-influence of the many factors that impact this experience [14]. Each woman experiences her care differently depending on her contexts of vulnerability and the factors that influence her. With this in mind, prenatal nursing care must favor a woman-centered approach [28] that is holistic, individualized, respectful, and that promotes women’s empowerment [29]. Taking a holistic approach means considering the woman as a whole, including biological, psychosocial and spiritual aspects [29]. According to El-Haddad et al. [30], women’s needs and expectations are directed toward the nurse, the prenatal care organization and the benefits they will derive from the care. However, our findings indicate that it is necessary to take into account all vulnerability and family contexts as they influence women’s prenatal care-related needs and expectations. Providing holistic care enables the nurse to better understand how a pregnancy affects the entire person and how to respond to the person’s actual needs [29].

An individualized approach requires providing the woman with personalized care that takes into account her unique personality, history and perspective [29]. This uniqueness is reflected in the intersectionality of the different vulnerability contexts, the intersection of several contexts related to the woman’s unique situation that may explain this vulnerability [31]. The women’s experience is also influenced by their relationship with the nurse. The nurse must opt for a respectful approach “to create a supportive relationship and to develop women’s knowledge, skills, power within oneself, and self-determination” [32].

Empowerment promotes the self-attitude and autonomy of women in contexts of vulnerability [29]. The nurse can foster women’s engagement and empowerment by giving them the power to decide on the content, the duration of the meetings, the setting, the objectives of the intervention plan or the frequency of the meetings, as they request. The woman’s involvement in decision-making is essential to enabling her to exercise some control over her prenatal care. This involvement also contributes to more woman-centered care, thus promoting a positive care experience [33, 34]. Vedam et al. [35] demonstrated that pregnant women in contexts of vulnerability may have limited decision-making power. This is reflected in their struggle to express their needs and expectations or in their low level of involvement in care-related decisions as they seek to conform to the existing care structure offered by the programs.

Whether or not they present contexts of vulnerability, during their prenatal care, all women have needs and expectations related to the clinician, the organization of care or to their personal circumstances [14, 36]. However, it is the complexity of this experience that differs for those in contexts of vulnerability as these contexts impact their entire prenatal care experience. For example, for an immigrant woman, language barriers and cultural differences add to the other usual pregnancy-related concerns and complicate the care experience. Similarly, being isolated and not receiving the support of their social circle, combined with problems in their family, school or married life will represent a major challenge for these women. Women in contexts of vulnerability will encounter more obstacles to a positive prenatal care experience [36], such as impersonal care, discrimination and receiving inadequate information.

### Clinical implications

The aim of the proposed conceptual framework is to support nursing practice in the promotion of a positive prenatal nursing care experience for women, as suggested by the WHO [6]. The results highlight the fact that during prenatal care, the nurse can make the women’s experience easier by considering the dimensions and factors proposed in the conceptual framework.

One of the key activities that should be prioritized consists in taking greater account of pregnant women’s contexts of vulnerability. The program regularly takes women’s financial difficulties into account by providing food vouchers. However, it is also important to consider other contexts, such as low health literacy, social isolation or the presence of a health issue. For example, the nurse can support a woman who doesn’t know how to complete the parental benefit forms that will help her receive an income during her pregnancy or help the woman get involved in organizations to reduce her isolation. Contexts of vulnerability expose women to inequity [15]. Thus, the nurse must promote equity in care [3, 28] through interventions that are specific to each woman’s different contexts of vulnerability. When caring for a woman living with financial difficulties and dysphasia, nursing interventions must take both of these contexts into account.

### Limitations of the study

The study was conducted in the province of Quebec. The number of local community service centers included in the study (*n* = 11) enabled the observation of a variety of experiences, namely with regard to geographical context (*n* = 5 regions of Quebec). The same can be said for the variability in the contexts of vulnerability and in the age of participants (16 to 39 years old). Finally, the women’s experiences varied greatly based on prenatal nursing care characteristics, namely setting, frequency, number of meetings, number of providers, duration of meetings [16]. The in-depth description of pregnant women’s different contexts may allow to transfer the findings to similar contexts. However, results are not applicable to hospital care, high-risk pregnancies or follow-up by health professionals other than the nurse.

Other healthcare providers contribute to prenatal care, such as physicians, midwives, and gynecologists. This study focused on nursing care because the prenatal care in Olo and SIPPE programs is provided by nurses.

The study was conducted against the backdrop of the global COVID-19 pandemic, which may have influenced the pregnant women’s statements and contexts. This may have contributed to the fact that several women reported being socially isolated or having been imposed the setting of the prenatal care.

The results suggest that there is a need for future research to better understand the influence of certain factors and to study other contexts of vulnerability. As explained above, many vulnerability contexts have been individually studied in depth as part of other studies. Here, we present an overview of the experience of pregnant women who may be experiencing more than one context, as shown in our results. However, in this study, some contexts were not included, such as homeless pregnant women or those with a judicial record. The judicial record could add other factors, such as the influence of correctional officers or delays in communication with the nurses [37]. The findings of the study by McGeough et al. [38] on pregnant women experiencing homelessness reached the same conclusion as our study. Further research could focus on this population and could validate the proposed conceptual framework. Due to the inclusion and exclusion criteria, immigrant women who did not speak French were excluded. However, one of the participants did fit this context.

## Conclusions

The prenatal nursing care experience of women in contexts of vulnerability is complex. This complexity stems from the many factors influencing the experience as well as the cross-influence of these factors. It also depends on each woman’s specific contexts of vulnerability, which affect each of the factors, thus adding an additional challenge for nurses. A woman-centered approach is necessary to properly address the multidimensionality of the factors influencing the experience. The nurse must adopt a woman-centered approach and foster the woman’s empowerment in order to improve health outcomes and ensure the equity of available services. By taking into account each of the factors influencing the woman’s experience, the nurse can contribute to a positive prenatal care experience, which will increase these women’s utilization of prenatal services, and ultimately improve health outcomes for this population.

## Supplementary Information

**Additional file 1.** 

**Additional file 2.** 

**Additional file 3.** 

**Additional file 4.**

## Data Availability

The data that support the findings of this study are available from corresponding author, but restrictions apply to the availability of these data, which were used under license for the current study, and so are not publicly available. Data are however available from the corresponding author upon reasonable request and with permission of Émilie Hudon.

## Abbreviations

HIV: Human immunodeficiency virus
LCSC: Local Community Service Centre
LGBTQ2 +: Lesbian, gay, bisexual, transgender, queer (or sometimes questioning), and two-spirited
MD: Missing data
Olo: Eggs, milk and orange corresponding to Œufs-lait-orange
QUAGOL: Qualitative Analysis Guide of Leuven
SIPPE: Integrated Perinatal and Early Childhood Services corresponding to Services intégrés en périnatalité et pour la petite enfance
Tel: Telephone
WHO: World Health Organization
YPS: Youth protection services
