# Supplementary material for: The experience of pregnant women in contexts of vulnerability of prenatal primary nursing care: a descriptive interpretative qualitative study
Source: BMC Pregnancy Childbirth. 2023 Mar 18;23:187. doi: 10.1186/s12884-023-05474-z (PMC10023312; doi:10.1186/s12884-023-05474-z)
Supplement: Supplementary file 1 — Additional file 1. [file 12884_2023_5474_MOESM1_ESM.docx]

**Additional File 1**

*Standards for Reporting Qualitative Research (SRQR)*

Title and abstract

| **Title** - Concise description of the nature and topic of the study Identifying the study as qualitative or indicating the approach (e.g., ethnography, grounded theory) or data collection methods (e.g., interview, focus group) is recommended | Page 1 line 2-3 |
| --- | --- |
| **Abstract** - Summary of key elements of the study using the abstract format of the intended publication; typically includes background, purpose, methods, results, and conclusions | Page 2 line 48-77 |

Introduction

| **Problem formulation** - Description and significance of the  problem/phenomenon studied; review of relevant theory and empirical work; problem statement | Page 4 lines 82-106 |
| --- | --- |
| **Purpose or research questio**n - Purpose of the study and specific objectives or questions | Page 5 lines 106-109 |

Methods

| **Qualitative approach and research paradigm** - Qualitative approach (e.g., ethnography, grounded theory, case study, phenomenology, narrative research) and guiding theory if appropriate; identifying the research paradigm (e.g., postpositivist, constructivist/ interpretivist) is also recommended; rationale** | Page 5 line 113-115 |
| --- | --- |
| **Researcher characteristics and reflexivity** - Researchers’ characteristics that may influence the research, including personal attributes, qualifications/experience, relationship with participants, assumptions, and/or presuppositions; potential or actual interaction between researchers’ characteristics and the research questions, approach, methods, results, and/or transferability | Page 7 lines 155-161 |
| **Context** - Setting/site and salient contextual factors; rationale** | Page 5 lines 125-140 |
| **Sampling strategy** - How and why research participants, documents, or events were selected; criteria for deciding when no further sampling was necessary (e.g., sampling saturation); rationale** | Page 5 lines 120-123 |
| **Ethical issues pertaining to human subjects** - Documentation of approval by an appropriate ethics review board and participant consent, or explanation for lack  thereof; other confidentiality and data security issues | Page 5 lines 115-117  Page 5 lines 123-124 |
| **Data collection methods** - Types of data collected; details of data collection procedures including (as appropriate) start and stop dates of data collection and analysis, iterative process, triangulation of sources/methods, and modification of procedures in response to evolving study findings; rationale** | Page 6 lines 142-163 |
| **Data collection instruments and technologies** - Description of instruments (e.g., interview guides, questionnaires) and devices (e.g., audio recorders) used for data collection; if/how the instrument(s) changed over the course of the study | Page 6 lines 142-163  Additional File 2  Additional File 3 |
| **Units of study** - Number and relevant characteristics of participants, documents, or events included in the study; level of participation (could be reported in results) | Page 8 lines 188-196  Table 2  Figure 1  Table 3 |
| **Data processing** - Methods for processing data prior to and during analysis, including transcription, data entry, data management and security, verification of data integrity, data coding, and anonymization/de-identification of excerpts | Page 8 lines 168-184 |
| **Data analysis** - Process by which inferences, themes, etc., were identified and developed, including the researchers involved in data analysis; usually references a specific paradigm or approach; rationale** | Page 8 lines 168-184 |
| **Techniques to enhance trustworthiness** - Techniques to enhance trustworthiness and credibility of data analysis (e.g., member checking, audit trail, triangulation); rationale** | Page 7 lines 161-163  Table 1 |

Results/findings

| **Synthesis and interpretation** - Main findings (e.g., interpretations, inferences, and themes); might include development of a theory or model, or integration with prior research or theory | Page 11 line 218-349  Figure 2  Additional File 4 |
| --- | --- |
| **Links to empirical data** - Evidence (e.g., quotes, field notes, text excerpts, photographs) to substantiate analytic findings | Page 11 line 218-349  Additional File 4 |

Discussion

| **Integration with prior work, implications, transferability, and contribution(s) to the field -** Short summary of main findings; explanation of how findings and conclusions connect to, support, elaborate on, or challenge conclusions of earlier scholarship; discussion of scope of application/generalizability;  identification of unique contribution(s) to scholarship in a discipline or field | Page 17 line 351-413 |
| --- | --- |
| **Limitations** - Trustworthiness and limitations of findings | Page 19 line 415-441 |

Other

| **Conflicts of interest** - Potential sources of influence or perceived influence on study conduct and conclusions; how these were managed | Page 23 line 491 |
| --- | --- |
| **Funding** - Sources of funding and other support; role of funders in data collection, interpretation, and reporting | Page 23 line 493-498 |
| **The rationale should briefly discuss the justification for choosing that theory, approach, method, or technique rather than other options available, the assumptions and limitations implicit in those choices, and how those choices influence study conclusions and transferability. As appropriate, the rationale for several items might be discussed together. | |
| **Reference:** <http://www.equator-network.org/reporting-guidelines/srqr/> | |
| O'Brien BC, Harris IB, Beckman TJ, Reed DA, Cook DA. Standards for reporting qualitative research: A synthesis of recommendations. Academic Medicine. 2014; 89(9). DOI: 0.1097/ACM.0000000000000388 | |
