# Supplementary material for: The experience of pregnant women in contexts of vulnerability of prenatal primary nursing care: a descriptive interpretative qualitative study
Source: BMC Pregnancy Childbirth. 2023 Mar 18;23:187. doi: 10.1186/s12884-023-05474-z (PMC10023312; doi:10.1186/s12884-023-05474-z)
Supplement: Supplementary file 2 — Additional file 2. [file 12884_2023_5474_MOESM2_ESM.docx]

**Additional File 2**

*Sociodemographic questionnaire*

1. **What is your date of birth?**

Day Month Year ___

1. **What is your birth place?**

Quebec

Other province of Canada

Other country, specify : _________________

1. **What is your ethnic origin?**

Aboriginal

Caucasian

Other, specify : _________________

1. **In which CLSC do you get your follow-ups?**

CLSC du CIUSSS du Saguenay-Lac-Saint-Jean

CLSC du CIUSSS de l’Estrie-CHUS

CLSC du CISSS de la Montérégie centre

Other, specify : _________________

1. **What is your native language?**

French

Other, specify : _________________

1. **What is the highest level of education you have completed?**

No formal schooling

Part of primary school (elementary)

Completed primary school (elementary)

Part of secondary school (high school)

Completed secondary school (high school)

Completed vocational training

CEGEP or technical college completed

University completed

1. **Which sentence best describes your current occupation?**

Employed (full-time or part-time, including self-employed or on a work training program)

Unemployed and looking for work

At school full-time or in full-time education

Unable to work due to long-term sickness or disability

Looking after home/family/maternity leave

Other, specify :___________________________________

1. **Compared to other people your age, how do you perceive your financial situation?**

I consider myself financially comfortable

I consider my income adequate to meet my needs or those of my family (housing, food, drugs, etc.)

I consider my income inadequate to meet my needs or those of my family (housing, food, drugs, etc.)

I consider my income very inadequate to meet my needs or those of my family (housing, food, drugs, etc.)

1. **Overall, is your total income from all sources before taxes and other deductions:**

Less than $10 000

$10 000 to $14 999

$15 000 to $19 999

$20 000 to $24 999

$25 000 to $29 999

$30 000 to $39 999

$40 000 to $49 999

$50 000 to $59 999

$60 000 to $74 999

$75 000 to $99 999

$100 000 and more

1. **Who do you live with?**

Alone

With your parents

With your children

With your spouse

With your spouse and children

Other, specify : __________________________________

1. **How many other children did you give birth to, excluding this pregnancy?** _________
2. **What is your marital status?**

Married, living with a partner

Separated, divorced

Widowed

Single

1. **Do you get support from others when you need it (e.g., friends or family)?**

No

Yes

1. **Are you pregnant?**

No (Do not complete questions 15 and 16)

Yes (Do not complete questions 17)

1. **What is the expected date of delivery**? Day Month Year____
2. **How many weeks have you been pregnant?** _______^/7^ weeks
3. **What was the expected date of delivery?** Day Month Year____
4. **Number of visits received to date from the CLSC nurse for your pregnancy follow-up? (**approximate) ___________________
5. **Which of the following services do you receive?** (You may check more than one answer)

Olo

SIPPE

Other, specify: _____________________________

1. **Is this the first time you have been followed by a SIPPE nurse?** (You may check more than one answer)

No

Yes

1. **How long do the meetings last?** (approximate) ___________________
2. **Where are the meetings held?**

Home

CLSC

Telephone

Other, specify: _____________________________

1. **How many nurses have you seen during your follow-up?** ___________________
2. **Do you have a physical or mental health problem? If yes, specify:**

______________________________________________________________________

Abbreviations. LCSC: local community service center; Olo: Œufs, lait, orange (Eggs, milk, orange); SIPPE: Services intégrés en périnatalité et pour la petite enfance (Integrated Perinatal and Early Childhood Services).
