# Supplementary material for: The experience of pregnant women in contexts of vulnerability of prenatal primary nursing care: a descriptive interpretative qualitative study
Source: BMC Pregnancy Childbirth. 2023 Mar 18;23:187. doi: 10.1186/s12884-023-05474-z (PMC10023312; doi:10.1186/s12884-023-05474-z)
Supplement: Supplementary file 3 — Additional file 3. [file 12884_2023_5474_MOESM3_ESM.docx]

**Additional File 3**

*Interview Guide*

**Opening**

- Please tell me about your prenatal nursing care experience.

**Question #1**

- What has led you to consult the LCSC nurses for your prenatal care?

**Question #2**

- Can you tell me about your prenatal care/nursing-related needs or expectations (e.g., occupation, work, home)?

**Question #3**

- Please tell me what you enjoy about the prenatal care.

**Question #4**

- Please describe what you enjoy the least about your prenatal care or your experience with the nursing staff.

**Question #5**

- Please describe how you feel during your meetings.
- Emotions
- Questions

**Question #6**

- Please explain why you feel this way during your meetings?

**Question #7**

- How would you describe your relationship with the nurse?
- What type of relationship is it?
- Please describe the impact of this relationship on your care.

**Question #8**

- What keeps you coming back for the care?

**Question #9**

- What change(s) could we make to your care to improve your experience?

**Closing**

- Is there anything else that you haven’t mentioned during the interview and that you would like to add? If so, please specify.

Abbreviations. LCSC: local community service center.
