# Supplementary material for: The experience of pregnant women in contexts of vulnerability of prenatal primary nursing care: a descriptive interpretative qualitative study
Source: BMC Pregnancy Childbirth. 2023 Mar 18;23:187. doi: 10.1186/s12884-023-05474-z (PMC10023312; doi:10.1186/s12884-023-05474-z)
Supplement: Supplementary file 4 — Additional file 4. [file 12884_2023_5474_MOESM4_ESM.docx]

**Additional File 4**

*Factors influencing th prenatal nursing care experience of women in contexts of vulnerability*

| **Dimensions** | **Factors** | **Examples** | **Verbatim** |
| --- | --- | --- | --- |
| **Fulfillment of pregnant women’s prenatal care needs and expectations** | Past experiences | Prenatal care at the LCSC | Whether I was going to connect with the person because this is something that is important […]. I was apprehensive […] about knowing how things were going to happen or knowing that it was someone from the LCSC since I’ve never really had a good relationship with people from the LCSC. (Sarah) |
|  |  | Previous pregnancy | If I had never given birth, I certainly I would have had more questions on how things happen. (Dalhia) |
|  |  | Prenatal nursing care | I had already been through it for my son in the past, so I knew what it was. I like my nurse very much, so I feel like seeing her again. (Ella) |
|  | Current pregnancy experience | New role as a parent | The nurse was very present, reassuring me about my role as a mother, as well as about becoming a parent. (Isabella) |
|  |  | Pregnancy signs, symptoms and complications | [The nurse] answered my questions about diabetes as well. She showed me how to inject myself with insulin and explained how to do it correctly. (Kiara) |
|  |  | Couple | We also had questions about sex; can it affect the baby? Things like that. (Héléna) |
|  |  | Newborn development | [The follow-up] was really to check on my baby. (Ella) |
|  |  | Relationship with the nurse | That’s what I needed from my nurse, a bond of trust. (Isabella) |
| **Fulfillment of pregnant women’s prenatal care needs and expectations** | Family concerns | Services for partner | [My spouse] is struggling with parenting, in terms of his self-confidence. […] I wish he could have had a follow-up with an outside person who is not… who is impartial […]. A social worker or a [psychologist] who could help him get through this and finally move it along, get it over with. When I found out [that this service was available through the Olo program], it was added along the way. (Aria) |
|  |  | Services for children | [The nurse] focuses a lot on my pregnancy and not on my son. (Flora) |
|  | Motivation | Recommendation from health professionals | Christina, who was receiving care for her second pregnancy, mentioned:  “[The nurse] said—normally, when we have follow-ups with [women in your situation] […]—I can’t see why the YPS would take the child from you because we’re [supporting you].”  This influenced her motivation to take part in the program:  “I didn’t take any chances since the YPS was involved in my daughter’s case. I said to myself—darn, if I have help with this child, like from the Olo program at the LCSC, [and] I’m taking part in the SIPPE program, well, maybe the YPS won’t come and take it away from me right away.” |
|  |  | Initial contact | [The nurse] had a good approach on the phone from the very beginning […]. I liked it, it made me want to go, to find out more about what her job is and what she could do for me. (Fernanda) |
|  |  | Social circle | It was my sister who recommended it to me [prenatal nursing care], because she had taken part [in the SIPPE and Olo programs], especially because of my financial situation, it helped me […], I knew I was going to get help. (Émilia) |
| **Fulfillment of pregnant women’s prenatal care needs and expectations** | Contexts of vulnerability | Limited financial resources | I needed more [help] in terms of being able to get proper groceries for the kids, for me and the baby, and then to be able to carry on with that. (Dalhia) |
|  |  | Health issue (dysphasia) | Because I suffer from dysphasia, I have a greater need to have someone with me so I really know what to do… Really do the right thing during my pregnancy. (Alya) |
|  |  | Health literacy | I have forms to fill out that I don’t understand. [The nurse] helped me answer the questions on the questionnaire […], as I needed a medical report for social assistance, for a pregnancy certificate. (Jenna) |
| **Pregnant women’s perception of nurses** | Nurse’s approach | Openness | If [the nurse] hadn’t been as open-minded as she was, I think I would have felt more like things were being imposed on me, and I would have enjoyed the meeting less. (Élisa) |
|  | Nurse’s characteristics | Age | Of course, the fact [that the nurse] is younger helps because when you’re young, it’s more reassuring to have someone who is closer to your age group. Of course, I am more comfortable speaking with my nurse, who must be 27–28, than with my nutritionist […], who, I would say, is closer to her late forties. It’s different. (Brenda) |
|  |  | Experience | She was a 23-year-old, with no kids, looking at me with a big smile and then, she was like—what do you want to talk about today? It was not easy to forge a bond. The nurse’s level of experience has to weigh a little in the balance. Having a nurse who is experienced, and who knows her stuff can help build trust (Félicia) |
|  | Nurse’s interventions | Consideration of expectations/needs | I didn’t feel [that the LCSC nurses] were forcing us or that they absolutely wanted us to check things on the list. That’s not true… The first nurse really insisted that I check as many things as possible [things that I need], yes, but I don’t need them (Aria) |
| **Pregnant women’s perception of nurses** |  | Consideration of contexts of vulnerability | [The nurse] provided resources for me to call or check out. Thanks to that nurse, I was able to get several things, […] such as social pediatric services in my city, telephone numbers to find out if physiotherapy or osteopathy were available for low-income individuals, lots of little things like that.   She took care of me because I’d had lumbar sprains (Lyvia) |
|  |  | Woman-centered assessment | There’s really someone who cares about you, and it’s really about how you are doing, because, often, when you’re pregnant, people just see the belly. […] It may sound silly, but it feels good. Yes, I’m pregnant and, yes, my friends and family have concerns, but that’s not all there is. [Camilla] is still here, it is not just about the belly (Camilla) |
|  |  | Counselling and education | [The nurse] could see that I was stressed, because she had told me about the baby blues […] When she started explaining the baby blues to me, I looked at her like a stranger, she told me, “No, it’s not that you [are going to have] the baby blues, it’s just to explain it to you because it’s very important that you know what it is” (Bonita) |
| **Pregnant women’s perception of prenatal primary care organization** | Modalities of care | Content of the care | [The nurses] were pushing to finish the meeting, to rush it. I told them that we were really tired, and that it would have been nice if we could stop the video and just resume another day. (Héléna) |
|  |  | Care delivery setting | My nurse was really kind, but I didn’t get to know her that much as we met over the phone; it’s not the same bond as face-to-face either. (Clara) |
|  |  | Making an appointment | It’s easy, if I want an appointment in two weeks, I can have one. I like that. (Brenda) |
|  | Continuity of nursing care | Changing nurses | It was stressful, because I hate change; it makes me very anxious. So changing nurses made me really scared. (Bonita) |
|  | Program services | Follow-up with other providers | After three meetings to discuss it [a follow-up for her son], [the nurses] told me that I might get something, but I haven’t heard from them yet. (Aria) |
|  |  | Number of providers | You meet so many people when you are pregnant, you have appointments, especially for high-risk pregnancies […], at some point, it becomes dizzying. So the fewer people you have to meet, the better. (Héléna) |

Abbreviations. YPS: Youth protection services; Olo: Œufs, lait, orange (Eggs, milk, orange); SIPPE: Services intégrés en périnatalité et pour la petite enfance (Integrated Perinatal and Early Childhood Services).
